# Association between circulating microRNAs 486, 146b and 15b and serum betatrophin levels in obese; type 2 diabetic and non-diabetic children

**DOI:** 10.1186/s12902-020-00628-y

**Published:** 2020-09-29

**Authors:** Khalid M. Mohany, Osamah Al rugaie, Osama Al-wutayd, Abdullah Al-Nafeesah, Tahia H. Saleem

**Affiliations:** 1 Department of medical biochemistry, College of Medicine, Assiut University, Assiut, Egypt; 2 Department of Basic Medical Sciences, Unaizah College of Medicine and Medical Sciences, Qassim University, Unaizah, Saudi Arabia; 3 Department of Family and Community Medicine, Unaizah College of Medicine and Medical Sciences, Qassim University, Unaizah, Saudi Arabia; 4 Department of Pediatrics, Unaizah College of Medicine and Medical Sciences, Qassim University, Unaizah, Saudi Arabia

**Keywords:** Childhood obesity, T2DM, Betatrophin, microRNA-486, microRNA-146b and microRNA-15b

## Abstract

**Background:**

This study tested the association between serum levels of microRNA-486, −146b and -15b and betatrophin in normal and obese children with/without type 2 diabetes mellitus (T2DM).

**Methods:**

the study included 120 children; divided into three groups: G1 (50 healthy), G2 (35 obese) and G3 (35 obese with T2DM). The levels of microRNA-486, 146b and 15b and serum betatrophin were measured by their corresponding methods.

**Results:**

serum microRNA-486, −146b, −15b and betatrophin levels were significantly high in G3 followed by G2 then G1 (*p* = 0.002, > 0.001, > 0.001, and > 0.001, respectively). Especially in G3, these levels correlated positively with the BMI percentile (r = 0.44, 0.58, 0.38, and 0.46, *p* = 0.007, > 0.001, 0.021, and 0.005, respectively), serum glucose (r = 0.56, 0.49, 0.82, 0.60, and 0.42, *p* > 0.001, 0.003, > 0.001, and > 0.001, respectively) and HbA1c% (r = 0.56, 0.39, 0.66, and 0.42, *p* > 0.001, 0.019, > 0.001, and 0.032, respectively) while, showed negative correlations with correlated with serum insulin levels (r = − 0.37, − 0.42, − 0.58, and − 0.41, *p* = 0.021, 0.012, > 0.001 and 0.013, respectively) and with serum C-peptide levels (r = − 0.76, − 0.50, − 0.35 and − 0.42, *p* > 0.001, 0.002, 0.036 and 0.011, respectively). Serum betatrophin levels correlated positively with microRNA-486, −146b and -15b levels in G2 (r = 0.35, 0.80, and 0.67, *p* = 0.036, > 0.001, and,> 0.001, respectively), and in G3 (r = 0.57, 0.36, and 0.38, *p* > 0.001, 0.029 and, 0.023, respectively).

**Conclusions:**

Circulating microRNA-486, 146b and 15b increase significantly in obese children with T2DM and these levels correlate positively with serum betatrophin levels. Further studies are required to test the role of targeting of these microRNAs and betatrophin in the timely management of obesity and/or T2DM in children.

## Background

Obesity is a global health hazard that is associated with a variety chronic diseases or syndromes such as hypertension, coronary artery diseases, diabetes mellitus and cerebral stroke [1, 2]. Due to the alarming increased number of obese children, the World Health Organization had considered the childhood obesity as a major confrontation of the current century [2]. Globally, more than 43 million children are either overweight or obese [3]. These conditions affect more than 30% of children in Saudi Arabia [4].

The increasing prevalence of childhood obesity is accompanied by a future risk of developing type II diabetes mellitus (T2DM) in adults [5, 6] or maturity onset diabetes of the young [7]. The actual mechanisms involved in the development of childhood obesity and its consequences are not completely understood which impedes the timely management of these complications [5]. Many studies were done aiming at finding a valid marker for early detection and treatment of obesity and consequences.

MicroRNAs are short non-coding RNA molecules (≈25 nucleotides in length) that influence many cellular aspects such as metabolism, cell growth, and programmed cell death [8, 9]. They negatively regulate mRNAs translation or enhance their degradation [9]. Abnormal expression of microRNAs participates in the pathogenesis of many adverse events such as obesity, dyslipidemias, impaired glucose tolerance and diabetes mellitus [5, 10–12]. The exact role of microRNAs in obese and/or diabetic children is still under investigations. MicroRNA-486, 146b and 15b are well-known contributors in the enhancement of preadipocyte growth and the development of obesity and insulin receptor down-regulation [5]. Also, they have a negative impact on the functions of beta cells of Langerhans, glucose uptake and/or insulin signaling pathway and so, may lead to the development of diabetes mellitus [5, 11].

Betatrophin (ANGPTL8; MW; 22KDa) is a hormone that has an important role in both glucose and lipid metabolism [13, 14]. Its expression occurs mainly in liver cells and adipose tissues [13]. Previous studies that tested the association of serum betatrophin levels with glucose levels, insulin resistance and other metabolic parameters in obese and/or adult subjects with T2DM, revealed controversial results [15–21]. So far, a small number of studies tested this association in children with obesity and/or T2DM [21].

The current study measured the relative expressions of circulating microRNAs 486, 146b and 15b and serum levels of betatrophin in obese children with/without T2DM and compared them with those in a healthy control group. Also, it evaluated the association between serum betatrophin levels and these microRNAs and their correlations with different metabolic and anthropometric parameters to support or exclude the therapeutic benefit of these microRNAs and betatrophin or their antagonists in the management of children with obesity and/or T2DM.

## Methods

The study protocol was evaluated and approved by Qassim University medical ethics committee (approval # 3366-mduc-2018-1-14-S). The study was done in the biochemistry unit, Unaizah College of medicine, Qassim University, KSA in cooperation with the pediatrics and diabetes outpatient clinics between August 2018 and April 2019. It included 120 children (80 males and 40 females), aged between 6 and 14 years. A written informed consent was taken from all study participants’ parents.

A complete medical history was taken, and clinical examinations were done for all participants. The heights and weights of the participants were collected by a trained nursing staff. The heights were considered after taking off the child’s shoes to the nearest 0.5 cm and the weights were measured to the nearest 100 g also after taking off the shoes and in light clothes. The children body mass index (BMI) was calculated by the equation: BMI $=\frac{Weightin\  Kg}{height\ (m2)}$. The child was considered of normal weight when his BMI was between the 5th and 85th percentile for age and obese when his BMI was < 95 percentile for age [4, 22].

The eligible children were divided into 3 groups. G1; included 50 healthy children, G2; included 35 obese non-diabetic children and G3; included 35 obese children who were known to have T2DM according to their previous investigation (blood glucose concentration ≥ 200 mg/dL in venous plasma, fasting blood glucose ≥126 mg/dL, two-hours blood glucose during oral glucose tolerance test ≥200 mg/dL or hemoglobin A1c (HbA1c) ≥ 6.5% for diagnosis of diabetes mellitus together with normal or high insulin and/or C-peptide levels) [23]. Children with T2DM Children in G3 were managed by life-style modifications ± metformin treatment.

The child was excluded from the study when his parents refused to participate or when the child presented with any confounding condition such as type 1 diabetes mellitus, an endocrine disease (e.g. thyroid disorders, Cushing), or a systemic disease (e.g. neurological, hepatic, renal or cardiovascular) [5].

Seven mL venous blood sample was collected from each participant after 10 h of an overnight fasting. Four mL was collected in tubes containing EDTA and centrifuged for 8 min in a cool centrifuge (3000 rpm) to collect the plasma. Two mL of them was used to measure HbA1c% by its corresponding kit. The other 2 mL was used to measure the microRNAs relative expressions was done as previously described by Cui et al. (2018) [5]. Briefly, the plasma was then taken and stored in aliquot at − 80 **°**C. From 500 μL plasma, the total RNA was extracted by a Trizol-based miRNA isolation (ThermoFisher scientific, USA, Cat. No:15596–018) according to the accompanied protocol. TaqMan® MicroRNA Reverse Transcription Kit (Applied Biosystems, CA, USA, Cat. No: 4366596) and High Capacity cDNA Reverse Transcription Kit (Applied Biosystems, CA, USA, Cat. No: 438814) were used for RT-qPCR from 5 ng total RNA samples using specific microRNAs 486, 146b and 15b stem-loop primers. The synthetic cel-miR39 was added the sera (10^− 4^ pmol/μL) prior the microRNAs isolation for their quantification control. To calculate the differences of expression level for each of the studied microRNAs among samples, the 2-^ΔΔCT^ method for relative expressions was used [5].

The remaining 3 mL of sample was centrifuged at 1200×g for 10 min then the sera were collected into labeled Eppendorf tubes and kept at − 80 °C till analysis of other parameters. Serum betatrophin levels were measured by Human C19orf80 (Betatrophin) ELISA Kit (MyBioSource, Inc. San Diego, CA, USA, Catalog No: MBS761140). Serum fasting glucose, total cholesterol, HDL-c, LDL-c and triacylglycerols were measured by using routine procedures in the outpatient clinics laboratory.

The data were collected and analyzed by SPSS version 25. The expression of the continuous variables was as mean ± SD. The comparison of means between the three studied groups was done using the one-way ANOVA. The correlations between microRNAs relative expressions, betatrophin and other variables were tested by Pearson correlations coefficient. The *p*-Value was considered significant when ≤0.05. The ROC was done to test the efficacies of the relative expressions of microRNA 486,146b and15b and serum betatrophin levels in differentiating obese non-diabetic children from obese children with T2DM.

## Results

The relative expressions of microRNAs 486, 146b and 15b and serum levels of betatrophin were significantly higher in G3 and G2 when compared with G1 and in G3 when compared with G2 (Table 1). Similar results were found regarding BMI percentile, the levels of serum glucose, insulin, C-peptide, HbA1c%, and triacylglycerol (Table 1).

**Table 1:** Comparison of different studied variables (mean ± SD) among G1, G2 and G3

	G1 (healthy, *n* = 50)	G2 (obese, *n* = 35)	G3 (obese with T2DM, n = 35)	***p***-Value			
				G1Vs G2 Vs G3	G2 Vs G1	G3 Vs G1	G3 Vs G2
**Age (years)**	9.31 ± 1.47	9 ± 1.77	9.59 ± 1.40	0.273	0.38	0.36	0.12
**Gender**							
• Male	31 (62%)	27 (77%)	22 (63%)	0.294	0.16	0.78	0.29
• Female	19 (38%)	8 (23%)	13 (37%)				
**BMI percentile**	81.0 ± 1.2	96.8 ± 1.6	96.7 ± 1.4	< 0.001	< 0.001	< 0.001	0.935
**Glucose (mg/dL)**	96.46 ± 8.78	101 ± 15.40	116.65 ± 18.76	0.006	0.08	0.004	0.04
**Insulin (μg/mL)**	10.16 ± 1.48	10.50 ± 0.69	11.20 ± 0.93	0.037	0.20	0.002	0.014
**C-Peptide (ng/mL)**	2.98 ± 0.43	3.13 ± 0.21	3.21 ± 0.28	0.007	0.02	0.008	0.09
**HbA1c (%)**	4.17 ± 0.21	4.85 ± 0.99	7.32 ± 2.62	< 0.001	< 0.001	< 0.001	< 0.001
**Cholesterol (mg/dL)**	188.79 ± 21.26	190.14 ± 26.75	196.45 ± 26.49	0.34	0.79	0.14	0.32
**HDLc (mg/dL)**	39.30 ± 1.82	36.91 ± 8.12	35.61 ± 10.40	0.06	0.09	0.04	0.55
**LDLc (mg/dL)**	127.43 ± 20.12	131.39 ± 25.95	135.72 ± 22.23	0.252	0.43	0.08	0.45
**TAG (mg/dL)**	110.32 ± 22.43	111.17 ± 18.56	125.61 ± 20.65	0.001	0.80	0.002	0.001
**miR-486R**	1.52 ± 0.38	2.11 ± 0.25	2.71 ± 0.29	0.002	0.003	< 0.001	0.001
**miR-146bR**	2.42 ± 0.58	3.01 ± 0.18	3.54 ± 0.24	< 0.001	< 0.001	< 0.001	< 0.001
**miR-15bR**	3.82 ± 0.56	4.32 ± 0.27	5.10 ± 0.36	< 0.001	< 0.001	< 0.001	< 0.001
**Betatrophin (ng/mL)**	47.17 ± 6.85	54.54 ± 17.67	71.68 ± 29.63	< 0.001	< 0.001	< 0.001	< 0.001

***SD*:** standard deviation, ***T2DM*** type 2 diabetes mellitus, ***BMI*** body mass index, ***HDLc*** high density lipoprotein cholesterol, ***LDLc*** low density lipoprotein cholesterol, ***HbA1c*** glycated hemoglobin, ***TAC*** triacylglycerol, ***microR-486R*** microRNA 486 relative expression, ***miR-146bR*** microRNA 146b relative expression, ***miR-15bR*** microRNA 15b relative expression.

### Correlations (Table 2, Figs. 1 & 2)

Correlation of circulating microRNAs relative expressions and serum betatrophin levels with the other studied parameters in the three groups is shown in Table 2.

**Table 2:** Correlation of circulating microRNAs relative expressions and serum betatrophin levels with the other studied parameters in the three groups

			BMI percentile	Cholesterol (mg/dL)	Glucose (mg/dL)	HDLc (mg/dL)	LDLc (mg/L)	HbA1c (%)	TAG (mg/dL)	Insulin (μg/mL)	C-Peptide (ng/mL)
**G1 (healthy, n = 50)**	**miR-486R**	r	0.282^*^	0.205	0.323^*^	0.078	0.167	0.271	0.190	−0.293^*^	− 0.176
		p	**0.048**	0.154	**0.022**	0.590	0.247	0.057	0.186	**0.039**	0.223
	**miR-146bR**	r	−0.056	−0.014	0.049	0.126	−0.043	−0.171	0.075	−0.135	− 0.156
		p	0.701	0.921	0.736	0.383	0.765	0.235	0.604	0.351	0.279
	**miR-15bR**	r	0.254	0.088	0.130	0.104	0.067	0.377^**^	0.073	−0.450^**^	−0.235
		p	0.075	0.546	0.368	0.472	0.645	**0.007**	0.616	**0.001**	0.100
	**Betatrophin (ng/mL)**	r	0.335^*^	−0.087	0.339^*^	− 0.196	− 0.013	0.316**	− 0.275	0.064	0.053
		p	**0.017**	0.548	**0.016**	0.172	0.930	**0.049**	0.063	0.661	0.713
**G2 (obese,** ***n*** **= 35)**	**miR-486R**	r	0.335^*^	0.073	0.691^**^	−0.510^**^	0.238	0.785^**^	−0.022	− 0.554^**^	− 0.424^*^
		p	**0.049**	0.675	**0.000**	**0.002**	0.168	**0.000**	0.902	**0.001**	**0.011**
	**miR-146bR**	r	0.673*	−0.288	0.397^*^	−0.362^*^	− 0.154	0.493^**^	− 0.205	− 0.314**	− 0.332*
		p	**0.000**	0.094	**0.018**	**0.033**	0.378	**0.003**	0.237	**0.046**	**0.041**
	**miR-15bR**	r	0.454*	−0.291	0.483^**^	−0.359^*^	− 0.152	0.551^**^	− 0.249	− 0.359^*^	− 0.344^*^
		p	**0.018**	0.090	**0.003**	**0.034**	0.383	**0.001**	0.150	**0.034**	**0.043**
	**Betatrophin (ng/mL)**	r	0.376^*^	−0.293	0.392^*^	−0.242	− 0.206	0.363*	− 0.146	− 0.237	−0.124
		p	**0.026**	0.087	**0.020**	0.161	0.236	**0.041**	0.403	0.171	0.579
**G3 (obese with T2DM,** ***n*** **= 35)**	**miR-486R**	r	0.446^**^	0.086	0.564^**^	0.125	0.032	0.561^**^	0.785^**^	−0.371*	− 0.765^**^
		p	**0.007**	0.621	**0.000**	0.473	0.855	**0.000**	**0.000**	**0.021**	**0.000**
	**miR-146bR**	r	0.588^**^	−0.077	0.495^**^	0.067	−0.127	0.395^*^	0.421^*^	−0.427**	− 0.502^**^
		p	**0.000**	0.661	**0.003**	0.703	0.466	**0.019**	**0.012**	**0.012**	**0.002**
	**miR-15bR**	r	0.388^*^	−0.019	0.824^**^	0.218	−0.102	0.669^**^	0.283	−0.583^**^	−0.355^*^
		p	**0.021**	0.912	**0.000**	0.209	0.558	**0.000**	0.099	**0.000**	**0.036**
	**Betatrophin (ng/mL)**	r	0.461^**^	0.077	0.601^**^	0.116	0.043	0.428*	0.458^**^	−0.410*	− 0.425^*^
		p	**0.005**	0.660	**0.000**	0.506	0.807	**0.032**	**0.006**	**0.013**	**0.011**

**Correlation is significant at the 0.01 level (2-tailed)

*Correlation is significant at the 0.05 level (2-tailed)

***T2DM*** type 2 diabetes mellitus, ***BMI*** body mass index, ***HDLc*** high density lipoprotein cholesterol, ***LDLc*** low density lipoprotein cholesterol, ***HbA1c*** glycated hemoglobin, ***TAC*** triacylglycerol, ***microR-486R*** microRNA 486 relative expression, ***miR-146bR*** microRNA 146b relative expression, ***miR-15bR*** microRNA 15b relative expression.

**Fig. 1:**
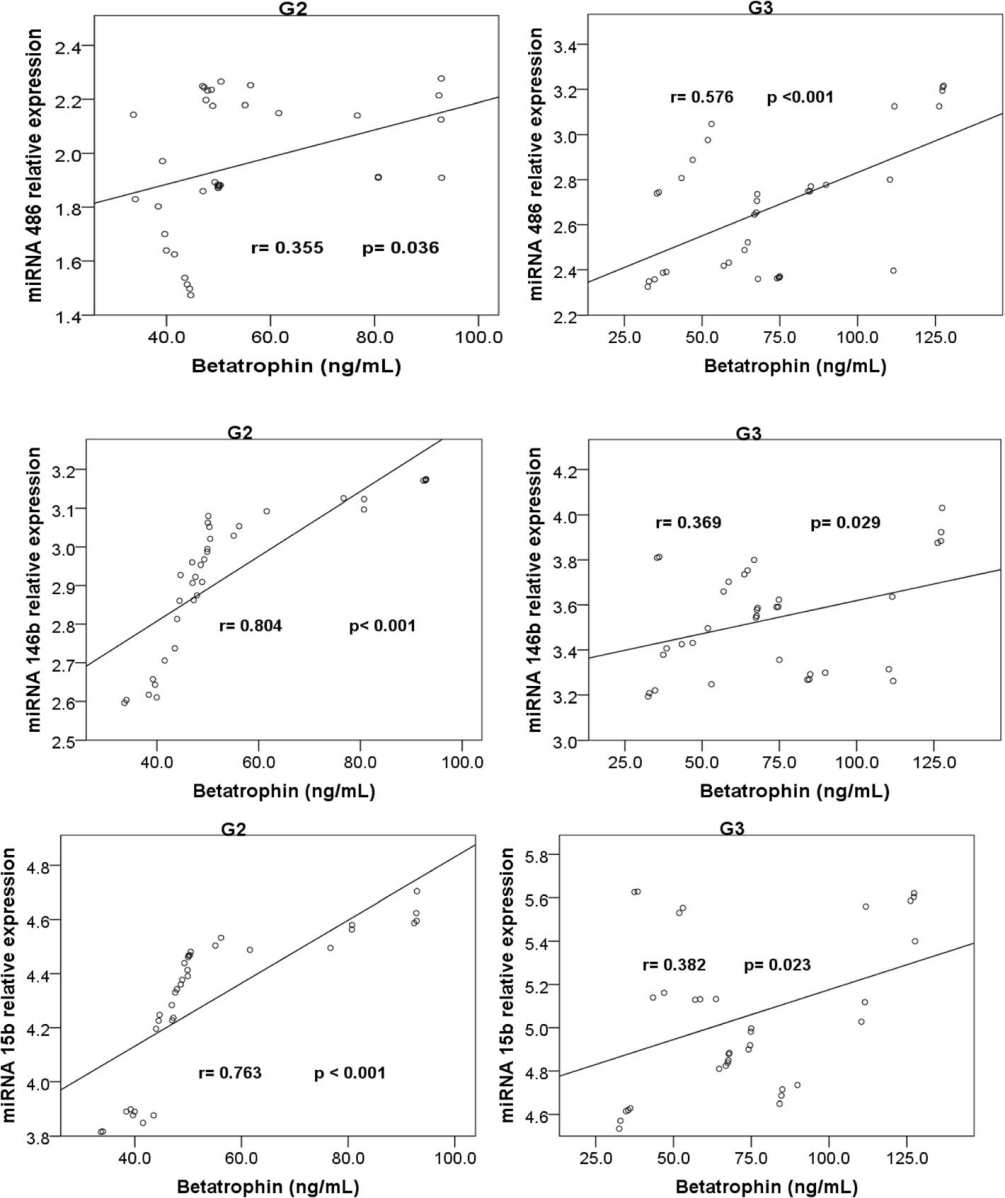
Correlations between serum betatrophin levels and microRNAs 486, 146b and 15b relative expressions in G2 and G3

**Fig. 2:**
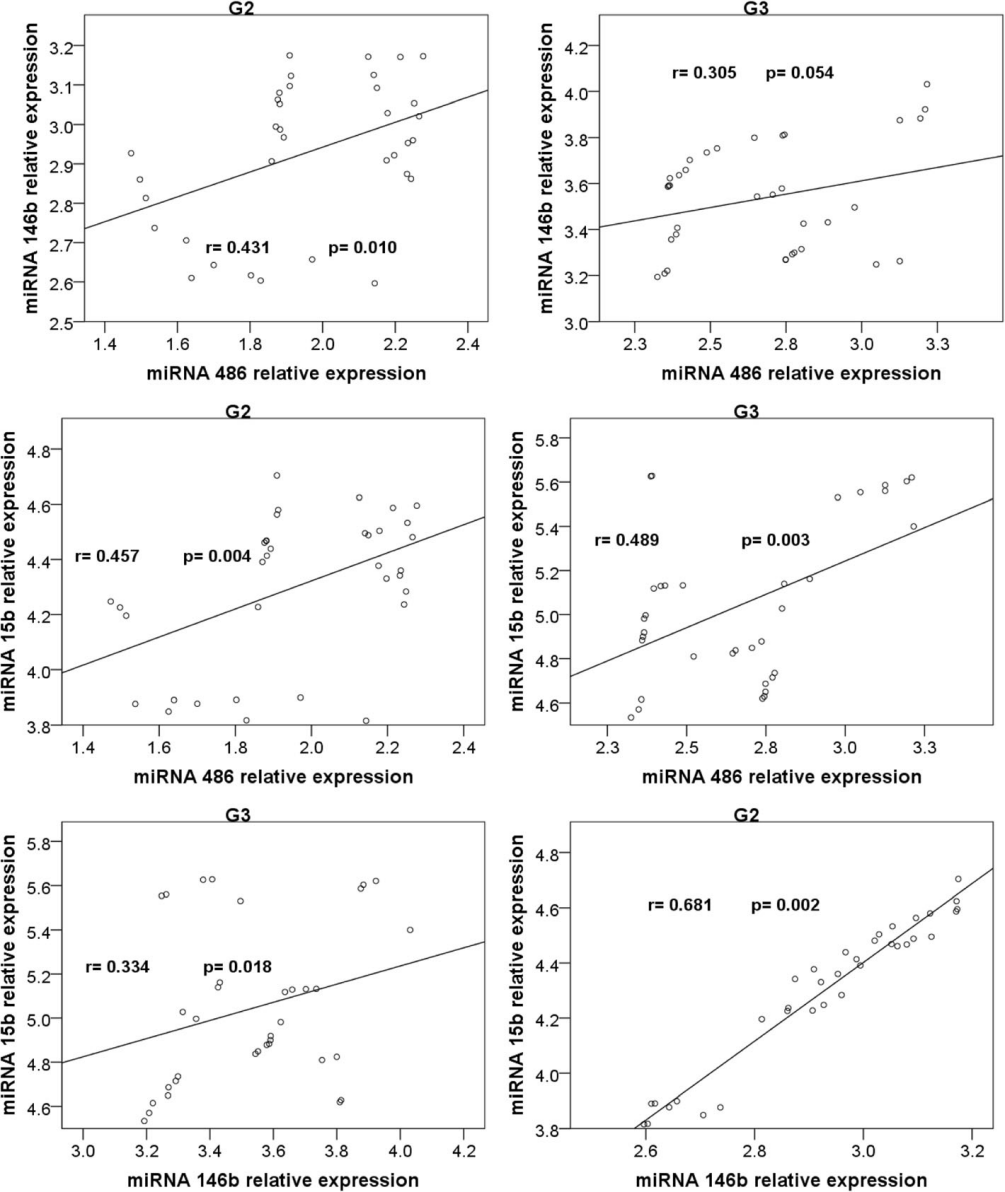
Correlations between the studied microRNAs relative expressions in G2 and G3

**miR-486R** showed significant positive correlations with BMI percentile and with fasting serum glucose levels (G1, G2 and G3), with HBA1c% (G2 and G3) and with triacylglycerols (G3). On the other hand, it showed significant negative correlations with HDLc (G2), with serum insulin levels (G1, G2 and G3) and with serum C-peptide levels (G2 and G3).

**miR-146bR** showed significant positive correlations showed significant positive correlations with BMI percentile, with fasting serum glucose levels and with HBA1c% (G2 and G3) and with triacylglycerols (G3). While, it showed significant negative correlations with HDLc (G2), with serum insulin levels and with serum C-peptide levels (G2 and G3).

**miR-15bR** showed significant positive correlations showed significant positive correlations with BMI percentile, and with fasting serum glucose levels (G2 and G3), and with HBA1c% (G1, G2 and G3). These relative expressions showed significant negative correlations with HDLc (G2), with serum insulin levels (G1, G2 and G3) and with serum C-peptide levels (G2 and G3).

**Serum betatrophin levels** showed significant positive correlations showed significant positive correlations with BMI percentile, with fasting serum glucose levels, and with HBA1c% (G1, G2 and G3) and with TAG levels (G3). On the other hand, these levels showed significant negative correlations with serum insulin levels and with serum C-peptide levels (G3).

The significant correlations among serum betatrophin levels and the relative expressions of miR-486, −146b and 15b in G2 and G3 are shown in Figs. 1 and 2. Non-significant correlations were noticed among them in G1.

**Fig. 3:**
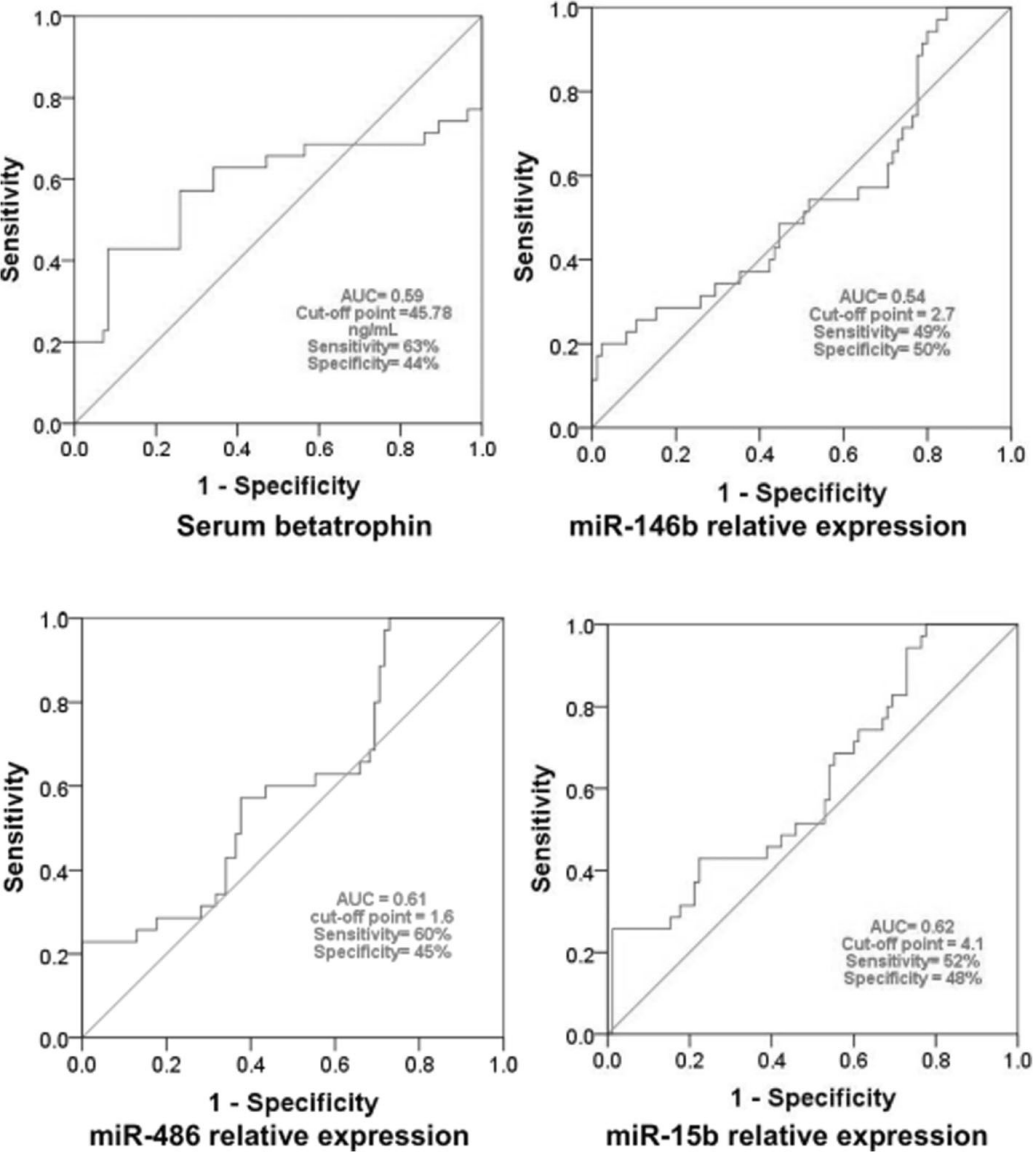
Efficacy of microRNAs relative expression and serum betatrophin in distinguishing non-diabetic obese children from obese children with T2DM. AUC: area under the ROC

The efficacy of circulating microRNAs 486, 146b and 15b relative expressions and serum betatrophin levels in differentiating non-diabetic obese children from obese children with T2DM is illustrated in Fig. 3. The areas under ROC were 0.61, 0.54, 0.62 and 0.59, respectively.

## Discussion

Childhood and adolescent obesity are escalating global health challenges with a growing risk of the development of T2DM in these age groups or in their adulthood [2, 3, 5–7]. The increased prevalence of childhood obesity was recognized mainly in developed and developing countries including the Saudi Arabia [4, 24]. Finding markers for early diagnosis and/or treatment of obesity and its complication was a target for many previous studies. So far, few studies had tested the role of microRNAs and betatrophin in obese children with/without T2DM.

MicroRNAs are small non-coding RNAs that regulates the gene expression post-transcriptionally either by preventing the translation of their targeted mRNAs or by degrading them [9]. They have a recognizable role in pathogenesis, prevention and diagnosis of many diseases including obesity and diabetes mellitus [5, 10–12]. In the current study, the circulating microRNA-486, −146b and -15b levels are found to be higher in obese children with/without T2DM than the healthy control. Also, the levels of these microRNAs correlate positively with the BMI, serum fasting glucose and HbA1c% especially in the obese children with/without T2DM. These findings may emphasize their important roles as metabolic regulators and in the pathogenesis of obesity and T2DM which is in accordance with the results of many previous studies [5, 25, 26]. White adipose tissue is a major source for these microRNAs in obese children [27]. MicroRNA-486 and 146b are well-known contributors in the enhancement of preadipocyte growth and the development of obesity [5, 26].

The positive correlations of these microRNAs with serum fasting glucose and HbA1c% and negative correlations with serum insulin levels (microRNA-486 and 15b) reflects their negative impact on the function of beta cells of Langerhans [5] and/or insulin signaling pathway (i.e. insulin resistance) [11]. MicroRNA-486 markedly decreases the glucose uptake by myotubes [5]. MicroRNA-146b affects the preadipocyte differentiation by targeting the Krueppel-like-factor-7 transcription factor. This factor has a role in the progress of T2DM either by the repression of insulin synthesis or by deregulation of adipocytokine synthesis in fat cells [27, 28]. Also, microRNA-146b promotes the myogenic differentiation in a way that may predispose to insulin resistance [29]. Yang et al. [2015] found an increased expression of microRNA-15b in obese mice and this was associated with insulin receptors down-regulation in the liver cells of models with T2DM [30]. The microRNA-15b binds the 3′ untranslated region of the insulin receptor mRNA preventing its translation and enhances its degradation which eventually leads to impairment in the insulin action i.e. insulin resistance [30].

Regarding the efficacies of the circulating microRNA486, 146b and 15b in differentiating children with T2DM from non-diabetic children, the areas under the ROC are much less in the current study (0.61, 0.54 and 0.62, respectively) when compared to the results of the study conducted by Cui et al. [2018] (0.923, 0.882 and 0.969, respectively) [5]. So, our results, in contrast to Cui et al. (2018), who suggest that the use of the relative expression of only one of these microRNAs is not satisfactory to differentiate children with T2DM from non-diabetic children. This may be attributed to the difference in the type and the size of the sample between the two studies. In the current study the subjects are only children and the sample size is 120 subjects; 35 of them are diabetic. While, in the aforementioned study, the subjects were both adults and children and the sample size was about 718 subjects, 101 of them are diabetic [5]. The use of a combination of more than one microRNA levels or their combination with serum betatrophin levels may increase the efficacy of them in distinguishing children with T2DM from non-diabetic children. This may be more logic if we consider the complex, multifactorial nature of the pathogenesis of diabetes mellitus.

Betatrophin is a peptide hormone that was wrongly proposed to increase the beta cell proliferation and mass expansion [13]. The treatment of the human beta cells of Langerhans with betatrophin had failed to induce their division which led to the retraction of this proposition later on [13, 31]. Betatrophin plays significant roles in both glucose and lipid metabolism [32]. Many studies had investigated betatrophin levels in obese and/or diabetic subjects and the results were controversial [15–21]. The current study finds significant higher levels of serum betatrophin in obese children with/without T2DM than the healthy group and these levels correlated positively with the BMI, serum fasting glucose and HbA1c%. These increased levels might be a compensation for the increased insulin requirement in the obese children with/without T2DM and are in agreement with findings of Fu et al. [2014] and Al-Daghri et al. [2016] [19, 22]. Also, Yamada et al. [2015] reported higher levels of serum betatrophin levels in cases with T2DMthan the healthy group [33]. Moreover, Abu-Farha et al. [2016] found increased serum betatrophin levels in obese individuals. These high levels declined markedly after 3 months of muscular exercise and so, they proposed a therapeutic role of reducing betatrophin in obese subjects [16].

On the other hand, the results of the present study are in contrast to the findings of Gomez-Ambrosi et al. [2014] who disclosed a decrease in serum betatrophin in obese individuals and this decrease was more evident when the obesity is associated with T2DM [18]. Also, the results of the current work are contradicting the results of Tuhan et al. [2016] who found lower levels of circulating betatrophin in obese individuals when compared to the healthy group and these levels correlated negatively with serum insulin levels [21]. In another study, Fenzl et al. [2014] found no correlation between serum betatrophin levels and blood glucose and insulin levels in obese adults [15].

## Conclusion

Circulating microRNA-486, 146b and 15b and serum betatrophin levels increase in obese children and this increase is much higher when the obesity is associated with T2DM. These levels correlate positively with the BMI centile, fasting serum glucose levels and glycosylated hemoglobin percentage while negatively correlated with serum insulin levels (486 and 15b). Further studies are required to test the role of targeting of these microRNAs and betatrophin in controlling obesity and/or T2DM in children.

## Data Availability

The datasets used and/or analyzed during the current study are available from the corresponding author on reasonable request.

## Abbreviations

RNA: Ribonucleic acid
T2DM: Type 2 diabetes mellitus
BMI: Body mass index
HbA1c: Glycosylated hemoglobin
WHO: World health organization
ANGPTL8: Angiopoietin-like protein 8
ELISA: Enzyme-linked immunosorbent assay
HDLc: High density lipoproteins cholesterol
LDLc: Low density lipoproteins cholesterol
EDTA: Ethylene diamine triacetic acid
ROC: The receiver operating characteristic

## References

[1] Al Dhaifallah A, Mwanri L, Aljoudi A (2015). Childhood obesity in Saudi Arabia: opportunities and challenge. Saudi J Obes.

[2] World Health Organization. Obesity: Preventing and managing the global epidemic. Report of a WHO consultation. WHO Technical Report Series. 2000; 894: 252. (World Health Organization, Geneva, 2000.) SFr 56.00, ISBN 92–4–120894-5. 11234459

[3] Ng M, Fleming T, Robinson M, Thomson B, Graetz N, Margono C (2014). Global, regional, and national prevalence of overweight and obesity in children and adults during 1980–2013: a systematic analysis for the global burden of disease study 2013. Lancet..

[4] World Health Organization (WHO): Country Cooperation Strategy for WHO and Saudi Arabia 2012–2016. (2013). Retrieved from: http://www.who.int/countryfocus/cooperation.strategy/ccs_sau_en.pdf.

[5] Cui X, You L, Zhu L, Wang X, Zhou Y, Wen J (2018). Change in circulating microRNA profile of obese children indicates future risk of adult diabetes. Metab Clin Exp.

[6] Kappil M, Wright RO, Sanders AP (2016). Developmental origins of common disease: epigenetic contributions to obesity. Annu Rev Genomics Hum Genet.

[7] Weintrob N, Stern E, Klipper-Aurbach Y, Phillip M, Gat-Yablonski G (2008). Childhood obesity complicating the differential diagnosis of maturity-onset diabetes of the young and type 2 diabetes. Pediatr Diabetes.

[8] Melkman-Zehavi T, Oren R, Kredo-Russo S, Shapira T, Mandelbaum AD, Rivkin N (2011). miRNAs control insulin content in pancreatic β-cells via down-regulation of transcriptional repressors. EMBO J.

[9] Sun W, Julie Li YS, Huang HD, Shyy JY, Chien S (2010). MicroRNA: a master regulator of cellular processes for bioengineering systems. Annu Rev Biomed Eng.

[10] Mohany KM, Rezk MY, Elkatawy HA (2017). Circulating miR-155 levels correlate with serum neutrophil Gelatinase associated Lipocalin in patients with diabetic nephropathy. IJDR..

[11] Feng J, Xing W, Xie L (2016). Regulatory roles of MicroRNAs in diabetes. Int J Mol Sci.

[12] Fischer-Posovszky P, Roos J, Kotnik P, Battelino T, Inzaghi E, Nobili V (2016). Functional significance and predictive value of MicroRNAs in pediatric obesity: tiny molecules with huge impact. Horm Res Paediatr.

[13] Yi P, Park JS, Melton DA (2013). Betatrophin: a hormone that controls pancreatic beta cell proliferation. Cell..

[14] Wang Y, Quagliarini F, Gusarova V, Gromada J, Valenzuela DM, Cohen JC (2013). Mice lacking ANGPTL8 (betatrophin) manifest disrupted triglyceride metabolism without impaired glucose homeostasis. Proc Natl Acad Sci U S A.

[15] Fenzl A, Itariu BK, Kosi L, Fritzer-Szekeres M, KautzkyWiller A, Stulnig TM (2014). Circulating betatrophin correlates with atherogenic lipid profiles but not with glucose and insulin levels in insulin-resistant individuals. Diabetologia..

[16] Abu-Farha M, Sriraman D, Cherian P, Elkum N, Behbehani K, Abubaker J, AlKhairi I (2016). Circulating ANGPTL8/Betatrophin is increased in obesity and reduced after exercise training. PLoS One.

[17] Al-Rawashdeh A, Kasabri V, Bulatova N, Akour A, Zayed A, Momani M (2017). The correlation between plasma levels of oxytocin and betatrophin in non-diabetic and diabetic metabolic syndrome patients: A cross sectional study from Jordan. Diab Metab Syndr.

[18] Gomez-Ambrosi J, Pascual E, Catalan V, Rodriguez A, Ramirez B, Silva C (2014). Circulating betatrophin concentrations are decreased in human obesity and type 2 diabetes. J Clin Endocrinol Metab.

[19] Fu Z, Berhane F, Fite A, Seyoum B, Abou-Samra AB, Zhang R (2014). Elevated circulating lipasin/betatrophin in human type 2 diabetes and obesity. Sci Rep.

[20] Wu S, Gao H, Ma Y, Fu L, Zhang C, Luo X (2016). Characterization of betatrophin concentrations in childhood and adolescent obesity and insulin resistance. Pediatr Diabetes.

[21] Tuhan H, Abaci A, Anik A, Catli G, Kume T, Calan OG (2016). Circulating betatrophin concentration is negatively correlated with insulin resistance in obese children and adolescents. Diabetes Res Clin Pract.

[22] Al-Daghri NM, Rahman S, Sabico S, Amer OE, Ansari MGA, Al-Attas OS (2016). Circulating betatrophin in healthy control and type 2 diabetic subjects and its association with metabolic parameters. J Diabetes Complicat.

[23] Reinehr T (2013). Type 2 diabetes mellitus in children and adolescents. World J Diabetes.

[24] Al SA (2015). Prevalence’s of overweight and obesity among Saudi children. IJSR..

[25] Sharma NK, Varma V, Ma L, Hasstedt SJ, Das SK (2015). Obesity associated modulation of mirna and co-regulated target transcripts in human adipose tissue of non-diabetic subjects. Microrna.

[26] Prats-Puig A, Ortega FJ, Mercader JM, Moreno-Navarrete JM, Moreno M, Bonet N (2013). Changes in circulating microRNAs are associated with childhood obesity. J Clin Endocrinol Metab.

[27] Chen L, Dai YM, Ji CB, Yang L, Shi CM, Xu GF (2014). miR-146b is a regulator of human visceral preadipocyte proliferation and differentiation and its expression is altered in human obesity. Mol Cell Endocrinol.

[28] Chakraborty C, Doss CG, Bandyopadhyay S, Agoramoorthy G (2014). Influence of miRNA in insulin signaling pathway and insulin resistance: micro-molecules with a major role in type-2 diabetes. Wiley Interdiscip Rev RNA.

[29] Khanna N, Ge Y, Chen J (2014). MicroRNA-146b promotes myogenic differentiation and modulates multiple gene targets in muscle cells. PLoS One.

[30] Yang WM, Jeong HJ, Park SW, Lee W (2015). Obesity-induced miR-15b is linked causally to the development of insulin resistance through the repression of the insulin receptor in hepatocytes. Mol Nutr Food Res.

[31] Jiao Y, LeLay J, Yu M, Naji A, Kaestner KH (2014). Elevated mouse hepatic betatrophin expression does not increase human β-cell replication in the transplant setting. Diabetes..

[32] Siddiqa A, Cirillo E, Tareen SH, Ali A, Kutmon M, Eijssen LM (2017). Visualizing the regulatory role of Angiopoietin-like protein 8 (ANGPTL8) in glucose and lipid metabolic pathways. Genomics..

[33] Yamada H, Saito T, Aoki A, Asano T, Yoshida M, Ikoma A (2015). Circulating betatrophin is elevated in patients with type 1 and type 2 diabetes. Endocr J.

